# Psychological resilience mediates the impact of anxiety and insomnia on health-related quality of life in maintenance dialysis patients

**DOI:** 10.1080/0886022X.2026.2693403

**Published:** 2026-07-09

**Authors:** Ruitong Liao, Xuemei Zhang, Lian Lin, Zhiyun Ning, Xiyan He, Yifan Lin, Yiqiang Zhan, Xiaoqing Ye

**Affiliations:** a Department of Epidemiology, School of Public Health (Shenzhen), Sun Yat-Sen University, Shenzhen, China; b Guangdong Engineering Technology Research Center of Nutrition Transformation, Sun Yat-sen University, Shenzhen, China; c The Seventh Affiliated Hospital, Sun Yat-sen University, Shenzhen, Guangdong, China; d Shenzhen Second People’s Hospital, Shenzhen, Guangdong, China; e The First Affiliated Hospital, Shenzhen University, Shenzhen, Guangdong, China

**Keywords:** Psychological resilience, anxiety, insomnia, quality of life, maintenance dialysis patients

## Abstract

This study examined the associations of anxiety, insomnia, and psychological resilience with health-related quality of life (HRQoL) among maintenance dialysis patients and explored potential mediating roles. In this cross-sectional study, 207 adult patients undergoing maintenance dialysis were recruited. HRQoL was assessed using the Short Form-36 Health Survey (SF-36), generating Physical Component Summary (PCS) and Mental Component Summary (MCS) scores. Anxiety, insomnia, and psychological resilience were measured using the Generalized Anxiety Disorder-7 (GAD-7), Athens Insomnia Scale (AIS), and 25-item Connor–Davidson Resilience Scale (CD-RISC-25), respectively. Multivariable linear regression and bootstrap-based mediation analyses were conducted. Furthermore, subgroup analyses were conducted to examine the robustness of the results.

Higher anxiety and insomnia scores were independently associated with lower PCS and MCS, whereas resilience was positively associated with both components. Each one-point increase in anxiety score was associated with a 1.61-point decrease in PCS and a 1.15-point decrease in MCS; corresponding decreases for insomnia were 1.01 and 0.91 points. Psychological resilience partially mediated the association between anxiety and HRQoL (23.46% for PCS; 30.37% for MCS), but no significant mediation was observed for insomnia. Subgroup analyses showed consistent associations for insomnia, while the anxiety–HRQoL relationship was modified by smoking status and diabetes. Anxiety and insomnia are independently associated with impaired HRQoL in dialysis patients. Psychological resilience partially explains the adverse impact of anxiety, but not insomnia, suggesting distinct patterns of association and potential targets for tailored psychosocial interventions.

## 1. Introduction

Chronic kidney disease (CKD) is a major global public health burden [1], affecting an estimated 850 million individuals worldwide, with approximately 4 million individuals requiring kidney replacement therapy due to kidney failure [2]. End-stage renal disease (ESRD) represents the irreversible, terminal phase of CKD, necessitating renal replacement therapy to sustain life, most commonly maintenance dialysis [3]. Maintenance dialysis patients generally bear a heavy disease burden and experience a significant impairment in health-related quality of life (HRQoL) [4,5], which has become a key patient-reported outcome in the comprehensive management of dialysis patients [6].

Health-related quality of life (HRQoL) is a multidimensional construct encompassing physical, psychological, and social well-being [7], and is commonly assessed using the Short Form-36 Health Survey (SF-36) questionnaire [8]. The decline in HRQoL among maintenance dialysis patients is very common, and it’s consistently associated with increased morbidity, hospitalization, and mortality [9]. Patients may exhibit impairments in both physical and mental HRQoL [10].

Psychological distress is highly prevalent among maintenance dialysis patients, and anxiety is one of the most commonly reported psychological symptoms [11]. A large-scale study including 61,497 hemodialysis patients and 4681 peritoneal dialysis patients showed that the prevalence of anxiety was 20.7% and 16.8%, respectively [12]. Previous studies have demonstrated that anxiety is significantly associated with poorer HRQoL in dialysis populations, particularly affecting mental health [13–15]. Sleep disturbances frequently co-occur with psychological distress in this population. Insomnia, as a typical sleep disorder, affects a substantial proportion of dialysis patients [16] and has been consistently linked to diminished HRQoL, particularly the physical health domains [17]. Therefore, it is particularly important to address the psychological issues of dialysis patients and promote their adaptation capacity.

Psychological resilience is the dynamic process whereby individuals maintain positive adaptation and effective functioning in the face of adversity, trauma, or significant stress [18]. Evidence showed that higher psychological resilience can alleviate the stress caused by diseases and dialysis treatments [19], and lead to better HRQoL [20]. Furthermore, psychological resilience may play a protective role against anxiety, depression [21], and sleep disturbances [22]. However, despite growing evidence supporting the beneficial role of psychological resilience, the interplay between resilience, psychological distress, and HRQoL in maintenance dialysis patients has not been fully elucidated.

Therefore, our study aimed to investigate the associations of anxiety, insomnia, and psychological resilience with health-related quality of life, as assessed by SF-36 PCS and MCS scores, among maintenance dialysis patients. In addition, we sought to explore whether psychological resilience mediates the relationships between anxiety, insomnia, and HRQoL. Understanding these relationships may provide insights into potential psychosocial intervention targets for improving quality of life among patients receiving long-term dialysis therapy.

## 2. Methods

### 2.1. Study design and population

This cross-sectional study was conducted between January and June 2025 at the Nephrology Center of a tertiary hospital in Guangdong Province, China. Adult patients receiving maintenance dialysis were consecutively recruited as the study population.

Eligible participants met the following inclusion criteria: (1) aged 18 years or older; (2) receiving maintenance dialysis therapy, including hemodialysis and peritoneal dialysis, for at least three months; and (3) being conscious and able to communicate effectively with the research staff and complete study questionnaires independently or with minimal assistance. Patients were excluded if they: (1) had a documented history of psychiatric disorders or cognitive impairment; or (2) were experiencing acute complications or unstable clinical conditions at the time of enrollment.

The sample size was determined based on methodological recommendations for multivariable regression analyses, which suggest including at least 10–15 participants per independent variable to ensure model stability and adequate statistical power. Considering the total number of independent variables (including primary exposure, mediator, and 10 covariates) in the fully adjusted models, a minimum sample size of approximately 180 participants was deemed sufficient. Anticipating a 20% nonresponse rate, we targeted a recruitment of 225 participants. During the study period, 227 eligible patients were approached, and a total of 207 valid samples were included in the final analysis.

To ensure data quality, all participants were enrolled using standardized procedures. Trained investigators administered questionnaires following a unified protocol, and completeness and consistency of the collected data were carefully checked.

The study protocol was approved and supervised by the Medical Ethics Committee of the Seventh Affiliated Hospital of Sun Yat-sen University (Shenzhen) (Approval No. KY-2025-110), and conducted in accordance with the Declaration of Helsinki principles. Written informed consent was obtained from all participants.

### 2.2. Covariates

Demographic, lifestyle, and clinical covariates were collected using a standardized questionnaire and through reviews of electronic medical records. Demographic characteristics included age (in years, as a continuous variable), sex (male/female), and educational level. Education was categorized into three levels: below high school, completed high school, and above high school. Marital status was also recorded and classified as married, unmarried, divorced, or widowed.

Lifestyle factors comprised body mass index (BMI), smoking status, drinking status, and physical exercise. BMI was calculated as weight in kilograms divided by height in meters squared (kg/m^2^). Smoking status was categorized as current smoker, former smoker, or never smoker. Similarly, drinking status was classified as current drinker, former drinker, or never drinker. Physical exercise was dichotomized as regular or not regular based on self-reported purposeful fitness activities at least three times per week.

Clinical characteristics included dialysis duration, hypertension, and diabetes. Dialysis duration (in months) was categorized into three groups: ≤12 months, 12–60 months, and ≥60 months. Both hypertension and diabetes mellitus were recorded based on self-reported history of physician diagnosis.

### 2.3. Health-related quality of life (SF-36)

Health-related quality of life (HRQoL) was assessed using the Chinese version of the Short Form-36 Health Survey (SF-36). Translated from the International Quality of Life Assessment (IQOLA) SF-36 Standard UK Version 1.0, this instrument was developed and validated in a Chinese general population [23], demonstrating satisfactory reliability and validity.

The SF-36 consists of 36 items grouped into eight domains: physical functioning (PF), role limitations due to physical problems (RP), bodily pain (BP), general health (GH), vitality (VT), social functioning (SF), role limitations due to emotional problems (RE), and mental health (MH). Each domain score is transformed to a scale ranging from 0 to 100, with higher scores indicating better health status [24].

In accordance with standard scoring procedures, two summary measures were calculated [8]: the Physical Component Summary (PCS) and the Mental Component Summary (MCS), which reflect overall physical and mental health status, respectively. PCS is primarily derived from PF, RP, BP, and GH domains, whereas MCS is mainly derived from VT, SF, RE, and MH domains. Both PCS and MCS were treated as continuous variables in the analyses.

### 2.4. Assessment of anxiety, insomnia, and psychological resilience

Anxiety was assessed using the Generalized Anxiety Disorder-7 (GAD-7) scale. The GAD-7 is a 7-item self-report questionnaire designed to evaluate the frequency of anxiety symptoms over the past two weeks. Each item is scored on a 4-point Likert scale ranging from 0 (“not at all”) to 3 (“nearly every day”), yielding a total score ranging from 0 to 21, with higher scores indicating greater anxiety severity. The Chinese version of the GAD-7 has demonstrated good reliability and validity in both general and clinical populations [25]. The GAD-7 was selected because it is a brief, well-validated screening tool widely used in clinical settings and has been successfully applied in patients with chronic kidney disease. In the present study, the total GAD-7 score was treated as a continuous variable in the regression and mediation analyses.

Insomnia was evaluated using the Athens Insomnia Scale (AIS) [26]. The AIS consists of eight items assessing nighttime sleep conditions and daytime functional status. Each item is rated on a 4-point scale (0–3), resulting in a total score ranging from 0 to 24. Higher scores indicate more severe insomnia symptoms. The validated Chinese version of the AIS has shown satisfactory psychometric properties in clinical settings. The scale was chosen because it provides a comprehensive assessment of both nocturnal sleep disturbances and daytime consequences, making it particularly suitable for evaluating insomnia in medically ill populations where daytime functioning is a key concern [27]. The total AIS score was analyzed as a continuous variable in this study.

Psychological resilience was measured using the 25-item Connor–Davidson Resilience Scale (CD-RISC-25) [28]. The Chinese version of the CD-RISC has been translated and validated, demonstrating good reliability and validity in diverse populations [29]. This instrument evaluates an individual’s ability to cope with adversity and adapt to stress. Each item is scored on a 5-point Likert scale ranging from 0 (“not true at all”) to 4 (“true nearly all the time”), yielding a total score between 0 and 100, with higher scores indicating greater resilience. The CD-RISC-25 was selected because it is a widely used and well-validated instrument for assessing psychological resilience in clinical and epidemiological research, including studies involving patients with chronic diseases. In this study, the total CD-RISC-25 score was treated as a continuous variable and examined as a mediator in the mediation analyses.

### 2.5. Statistical analysis

Continuous variables were presented as mean ± standard deviation (SD), and categorical variables were expressed as frequencies and percentages. To examine the associations of anxiety (GAD-7 score), insomnia (AIS score), and psychological resilience (CD-RISC-25 score) with HRQoL, multivariable linear regression models were constructed with PCS and MCS as continuous outcomes. Three progressively adjusted models were fitted: Model 1 was adjusted for age, sex, and education; Model 2 was further adjusted for BMI, smoking status, drinking status, and physical exercise; Model 3 was additionally adjusted for dialysis duration, hypertension, and diabetes mellitus. Regression coefficients (β) and 95% confidence intervals (CIs) were reported.

To investigate whether psychological resilience mediated the associations of anxiety and insomnia with HRQoL, mediation analyses were performed using the R package mediation. Separate mediation models were constructed for each exposure (GAD-7 score and AIS score) and outcome (PCS and MCS). Indirect effects, direct effects, total effects, and the proportion mediated were estimated using nonparametric bootstrap resampling with 1000 simulations. Due to the cross-sectional nature, the mediation analysis is exploratory and does not imply causal relationships.

Subgroup analyses were conducted to assess potential effect modification by sex, education, physical exercise, smoking status, drinking status, dialysis duration, hypertension, and diabetes. These analyses were conducted to examine whether the observed associations differed by demographic, lifestyle, and clinical characteristics. All statistical analyses were performed using R software (version 4.5.1). A two-sided *p* value <0.05 was considered statistically significant.

## 3. Results

### 3.1. Baseline characteristics of the participants

A total of 207 participants were included in the analysis, of whom 70 (33.8%) were female, and 137 (66.2%) were male (Table 1). The mean age of the study population was 49.92 ± 13.65 years. Most participants were married (78.3%) and had completed high school or above (57.5%).

**Table 1.** Baseline characteristics of patients stratified by sex.

Characteristic	*N*	Overall, %	Female	Male
N	207		70 (33.8)	137 (66.2)
Age, year	207	49.92 (13.65)	48.42 (16.34)	50.68 (12.04)
Education (%)	207			
Above high school		48 (23.2)	8 (11.4)	40 (29.2)
Below high school		88 (42.5)	44 (62.9)	44 (32.1)
Completed high school		71 (34.3)	18 (25.7)	53 (38.7)
Marital status (%)	207			
Divorced		9 (4.3)	4 (5.7)	5 (3.6)
Married		162 (78.3)	48 (68.6)	114 (83.2)
Unmarried		26 (12.6)	11 (15.7)	15 (10.9)
Widowed		10 (4.8)	7 (10.0)	3 (2.2)
BMI, kg/m²	207	23.49 (3.87)	22.23 (4.15)	24.13 (3.57)
Smoking status (%)				
Current		23 (11.1)	0 (0.0)	23 (16.8)
Former		54 (26.1)	2 (2.9)	52 (38.0)
Never		130 (62.8)	68 (97.1)	62 (45.3)
Drinking status (%)	207			
Current		5 (2.4)	0 (0.0)	5 (3.6)
Former		58 (28.0)	4 (5.7)	54 (39.4)
Never		144 (69.6)	66 (94.3)	78 (56.9)
Physical exercise (%)	207			
Regular		116 (56.04)	42 (60.0)	74 (54.0)
Not regular		91 (43.96)	28 (40.0)	63 (46.0)
Hypertension (%)	207			
Yes		172 (86.0)	51 (76.1)	121 (91.0)
No		28 (14.0)	16 (23.9)	12 (9.0)
Diabetes Mellitus (%)	207			
Yes		67 (33.7)	14 (20.3)	53 (40.8)
No		132 (66.3)	55 (79.7)	77 (59.2)
Dialysis duration, month	207			
≤12 months		54 (26.1)	16 (22.9)	38 (27.7)
12–60 months		111 (53.6)	36 (51.4)	75 (54.7)
≥60 months		42 (20.3)	18 (25.7)	24 (17.5)
GAD-7 total score	207	6.16 (4.29)	6.17 (4.42)	6.17 (4.26)
AIS total score	207	9.49 (5.06)	10.06 (5.30)	9.26 (4.96)
CD-RISC-25 total score	207	53.23 (17.18)	57.39 (19.31)	56.27 (21.85)
Physical component summary	207	59.34 (16.82)	59.30 (17.78)	59.36 (16.38)
Mental component summary	207	48.14 (15.15)	50.87 (16.61)	46.74 (14.21)

Data are presented as *n* (%) or mean ± SD.

BMI: Body Mass Index; GAD-7: Generalized Anxiety Disorder-7; AIS: Athens Insomnia Scale; CD-RISC: Connor–Davidson Resilience Scale.

The mean body mass index (BMI) was 23.49 ± 3.87 kg/m^2^. A majority of participants were never smokers (62.8%) and never drinkers (69.6%), while 56.0% reported engaging in regular physical exercise. Hypertension was highly prevalent (86.0%), and 33.7% of participants had diabetes. More than half of the patients (53.6%) had been undergoing dialysis for 12 to 60 months.

Regarding psychological measures, the mean GAD-7 total score was 6.16 ± 4.29, the mean AIS total score was 9.49 ± 5.06, and the mean CD-RISC-25 total score was 53.23 ± 17.18. The mean PCS and MCS scores were 59.34 ± 16.82 and 48.14 ± 15.15, respectively.

### 3.2. Associations of anxiety, insomnia, and psychological resilience with health-related quality of life

Table 2 presents the associations of anxiety, insomnia, and psychological resilience with the physical (PCS) and mental (MCS) components of health-related quality of life. For PCS, higher anxiety scores were significantly associated with lower PCS scores across all models. In the fully adjusted model (Model 3), each one-point increase in anxiety score was associated with a 1.61-point decrease in PCS (*β* = −1.61, 95%CI: −2.13 to −1.09, *p* < 0.001). Similarly, insomnia severity was independently associated with reduced PCS (*β* = −1.01, 95%CI: −1.48 to −0.54, *p* < 0.001). In contrast, psychological resilience was positively associated with PCS, with each one-point increase in CD-RISC-25 score corresponding to a 0.45-point increase in PCS (*β* = 0.45, 95%CI: 0.31 to 0.58, *p* < 0.001).

**Table 2.** Associations of anxiety, insomnia, and psychological resilience with health-related quality of life.

Variable	Model 1	*p* Value	Model 2	*p* Value	Model 3	*p* Value
**PCS**						
GAD-7 score	−1.59 (−2.08, −1.10)	**<0.001**	−1.51 (−1.98, −1.03)	**<0.001**	−1.61 (−2.13, −1.09)	**<0.001**
AIS score	−1.03 (−1.48, −0.59)	**<0.001**	−0.94 (−1.38, −0.51)	**<0.001**	−1.01 (−1.48, −0.54)	**<0.001**
CD-RISC-25 score	0.43 (0.30, 0.55)	**<0.001**	0.41 (0.28, 0.53)	**<0.001**	0.45 (0.31, 0.58)	**<0.001**
**MCS**						
GAD-7 score	−1.13 (−1.59, −0.68)	**<0.001**	−1.07(−1.52, −0.61)	**<0.001**	−1.15 (−1.64, −0.65)	**<0.001**
AIS score	−0.90 (−1.30, −0.50)	**<0.001**	−0.84 (−1.24, −0.45)	**<0.001**	−0.91 (−1.34, −0.48)	**<0.001**
CD-RISC-25 score	0.38 (0.26, 0.49)	**<0.001**	0.37 (0.26, 0.48)	**<0.001**	0.39 (0.26, 0.51)	**<0.001**

Data are presented as unstandardized regression coefficients (*β*) with 95% confidence intervals. Bold values indicate statistical significance at *P* < 0.05.

Model 1 adjusted for age, sex, and education.

Model 2 further adjusted for BMI, smoking status, drinking status, and physical exercise.

Model 3 additionally adjusted for dialysis duration, hypertension, and diabetes.

PCS: Physical Component Summary; MCS: Mental Component Summary; GAD-7: Generalized Anxiety Disorder-7; AIS: Athens Insomnia Scale; CD-RISC-25: 25-item Connor–Davidson Resilience Scale.

Comparable patterns were observed for MCS. In Model 3, anxiety was inversely associated with MCS (*β* = −1.15, 95%CI: −1.64 to −0.65, *p* < 0.001), and insomnia was also negatively associated with MCS (*β* = −0.91, 95%CI: −1.34 to −0.48, *p* < 0.001). Psychological resilience showed a significant positive association with MCS (*β* = 0.39, 95%CI: 0.26 to 0.51, *p* < 0.001). The magnitude and direction of associations were consistent across all Models, indicating the robustness of the findings after adjustment for demographic, lifestyle, and clinical covariates.

### 3.3. Mediation analyses

Mediation analyses were conducted to examine whether psychological resilience mediated the associations between anxiety, insomnia, and HRQoL (Table 3). The mediation analysis revealed that psychological resilience significantly mediated the relationship between anxiety and HRQoL. For PCS, the indirect effect (*β* = −0.38, 95%CI: −0.65 to −0.17, *p* < 0.001) accounted for 23.5% of the total effect. For MCS, the indirect effect (*β* = 0.35, 95%CI: −0.60 to −0.15, *p* < 0.001) accounted for 30.4% of the total effect. The direct effects of anxiety on both PCS and MCS also remained significant (*p* < 0.001), indicating partial mediation.

**Table 3.** Mediation effects of psychological resilience on the associations of anxiety and insomnia with health-related quality of life.

Variable	Outcome	Indirect effect		Direct effect		Total effect		% mediated
		*β* (95%CI)	*p* Value	*β* (95%CI)	*p* Value	*β* (95%CI)	*p* Value	
GAD-7 score	PCS	−0.38 (−0.65, −0.17)	**<0.001**	−1.23 (−1.88, −0.66)	**<0.001**	−1.61 (−2.30, −1.03)	**<0.001**	23.46%
	MCS	−0.35 (−0.60, −0.15)	**<0.001**	−0.80 (−1.40, −0.33)	**<0.001**	−1.15 (−1.80, −0.64)	**<0.001**	30.37%
AIS score	PCS	−0.14 (−0.36, 0.06)	0.174	−0.87 (−1.36, −0.39)	**<0.001**	−1.01 (−1.51, −0.53)	**<0.001**	14.21%
	MCS	−0.12 (−0.32, 0.07)	0.208	−0.78 (−1.22, −0.36)	**<0.001**	−0.91 (−1.37, −0.45)	**0.002**	13.69%

Data are presented as unstandardized regression coefficients (*β*) with 95% confidence intervals (CI). Bold values indicate statistical significance at *P* < 0.05.

All models were adjusted for age, sex, education, BMI, smoking status, drinking status, physical exercise, dialysis duration, hypertension, and diabetes.

PCS: Physical Component Summary; MCS: Mental Component Summary; GAD-7: Generalized Anxiety Disorder-7; AIS: Athens Insomnia Scale.

In contrast, psychological resilience did not significantly mediate the associations between insomnia and HRQoL. The indirect effects of insomnia on PCS (*β* = −0.14, 95%CI: −0.36 to 0.06, *p* = 0.174) and MCS (*β* = −0.12, 95%CI: −0.32 to 0.07, *p* = 0.208) were not statistically significant. However, the direct effects of insomnia on both PCS (*β* = −0.87, 95%CI: −1.36 to −0.39, *p* < 0.001) and MCS (*β* = −0.78, 95%CI: −1.22 to −0.36, *p* < 0.001) remained significant, as were the total effects. These findings suggest that the association between insomnia and HRQoL was primarily explained by the direct effect rather than through psychological resilience.

### 3.4. Subgroup analyses

**Figure 1.**
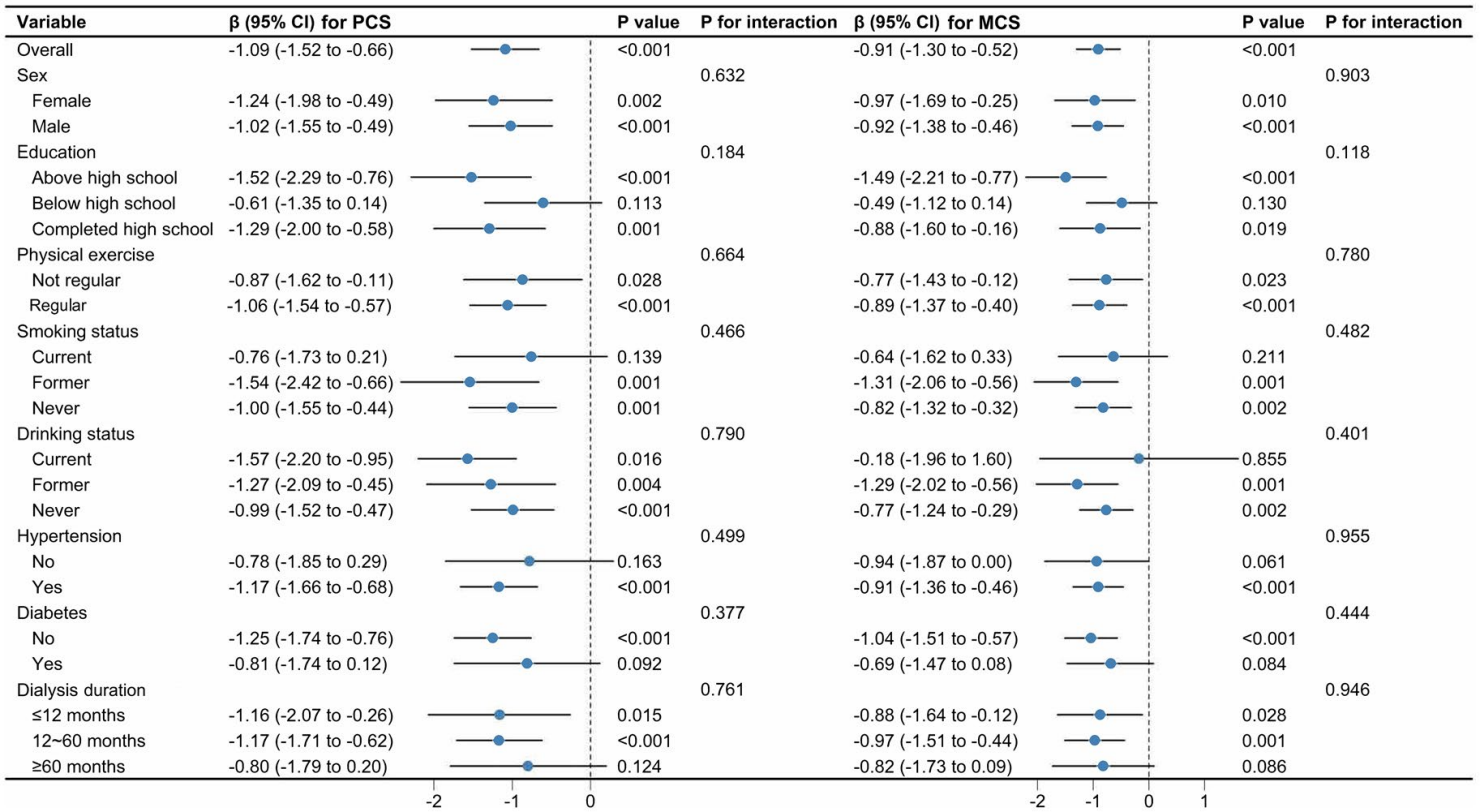
**Subgroup analyses of the association between insomnia and health-related quality of life**. Adjusted *β* coefficients and 95% confidence intervals (CIs) were estimated from multivariable models including sex, education, physical exercise, smoking status, drinking status, hypertension, diabetes, and dialysis duration. The left panel shows Physical Component Summary (PCS) and the right panel shows Mental Component Summary (MCS).

Subgroup analyses revealed that the negative association between insomnia and HRQoL was robust across demographic and clinical strata, with no significant interaction effects (Figure 1). This indicates that insomnia exerts a relatively uniform detrimental effect in maintenance dialysis populations.

**Figure 2.**
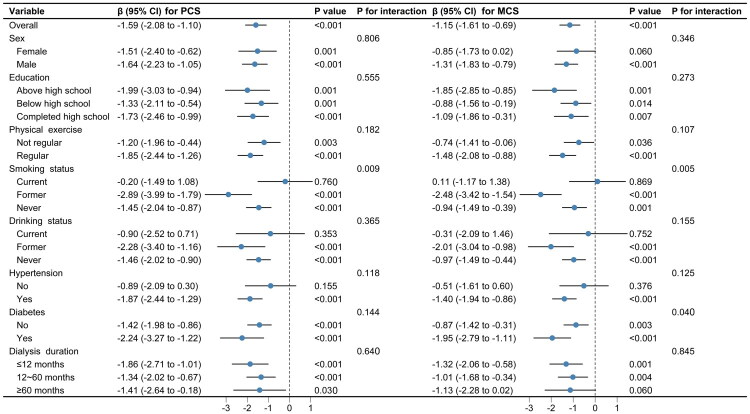
**Subgroup analyses of the association between anxiety and health-related quality of life**. Adjusted *β* coefficients and 95% confidence intervals (CIs) were estimated from multivariable models including sex, education, physical exercise, smoking status, drinking status, hypertension, diabetes, and dialysis duration. The left panel shows Physical Component Summary (PCS) and the right panel shows Mental Component Summary (MCS).

For anxiety, however, significant interaction effects were observed for smoking status (PCS and MCS) and diabetes (MCS only), whereas no significant effect modification was found for other variables (Figure 2).

## 4. Discussion

In this cross-sectional study of maintenance dialysis patients, we demonstrated that both anxiety and insomnia were significantly associated with poorer HRQoL, characterized by lower PCS and MCS scores. Psychological resilience partially mediated the association between anxiety and HRQoL, accounting for 23.46% and 30.37% of the total effects on PCS and MCS, respectively; in contrast, psychological resilience did not exert a significant mediating effect on the relationship between insomnia and HRQoL. These findings suggest that although both anxiety and insomnia are detrimental to HRQoL, their underlying pathways may differ.

Subgroup analyses further revealed that the inverse association between insomnia and HRQoL was robust across demographic and clinical strata, with no significant interaction effects. This indicates that insomnia exerts a relatively uniform detrimental effect in dialysis populations. By contrast, the association between anxiety and HRQoL was modified by smoking status (for both PCS and MCS) and by diabetes (for MCS only), suggesting that lifestyle factors and metabolic comorbidities may influence the strength of the association between anxiety and health outcomes.

Our findings are consistent with previous research demonstrating that anxiety and insomnia are highly prevalent among dialysis patients and are strongly associated with impaired HRQoL [13,15,16]. Emotional distress in this population has been linked not only to reduced mental well-being but also to poorer physical health perception [30]. Furthermore, psychological resilience has been increasingly recognized as a protective factor in chronic illness [31]. Previous studies in patients with chronic kidney disease have reported that higher psychological resilience levels are associated with better emotional adjustment and improved HRQoL [19,20]. Our study extends this literature by quantitatively demonstrating that psychological resilience functions as a partial mediator specifically in the anxiety–HRQoL pathway, thereby providing empirical support for a potential stress-adaptation pathway in which psychological resilience may act as a cognitive–emotional buffer linking anxiety to HRQoL.

In addition, evidence suggests that psychological resilience-enhancing interventions, including cognitive-behavioral therapy (CBT), mindfulness-based stress reduction, and supportive psychotherapy, can improve HRQoL, with particular benefits for mental health domains [32,33]. Notably, CBT is recommended as the first-line treatment for adult insomnia in clinical guidelines [34] and has demonstrated superior efficacy compared to pharmacotherapy [35], underscoring its potential to enhance HRQoL.

The observed partial mediation for anxiety supports a psychological stress-adaptation framework [36]. In maintenance dialysis patients, anxiety is often related to uncertainty about disease progression, treatment dependence, and financial or social burden [37]. Persistent anxiety may promote maladaptive cognitive appraisal and reduced self-efficacy, thereby weakening individuals’ capacity for adaptive coping. Lower resilience, in turn, may limit effective emotional regulation and stress management, leading to poorer perceived mental well-being and overall HRQoL. This finding aligns with prior evidence demonstrating that psychological resilience serves as a significant mediator between psychological symptoms and HRQoL among individuals with chronic diseases [38]. Furthermore, the larger mediation proportion observed for MCS than PCS suggests that psychological resilience may have a more pronounced influence on mental HRQoL than on physical functioning. This observation is consistent with theoretical and empirical work [39], indicating that resilience reflects effective emotion regulation and cognitive appraisal processes that are especially important for psychological adaptation to chronic illness and distress [40], whereas physical health outcomes are more directly influenced by physiological and disease-related factors.

Importantly, the conceptualization and expression of psychological resilience may be shaped by cultural context. In collectivist cultures, resilience often manifests through family cohesion, social support networks, and acceptance of illness as a shared experience; for patients undergoing long-term dialysis, reliance on familial caregiving and interpersonal relationships may reinforce adaptive coping and emotional regulation [41]. In contrast, in individualistic settings, resilience may be more closely linked to personal autonomy, active problem-solving, and self-reliance [42]. Cultural norms emphasizing perseverance and acceptance of hardship may also influence how individuals interpret illness-related stress and maintain psychological stability [43]. These cultural differences may partly explain variability in the role of resilience within the anxiety–HRQoL association and should be considered when interpreting findings across populations or designing culturally adapted interventions [44].

In contrast, the nonsignificant indirect effects in the insomnia models suggest that psychological resilience may not adequately buffer the impact of sleep disturbance on HRQoL. Insomnia may directly impair physical vitality and emotional functioning through persistent fatigue and neurobiological stress responses [45], irrespective of an individual’s level of psychological resilience. Therefore, interventions aimed at improving sleep may need to prioritize behavioral and physiological mechanisms over strategies focused solely on enhancing resilience.

Our findings possess several notable clinical implications for the management of maintenance dialysis patients. First, anxiety was associated with poorer HRQoL both directly and indirectly through lower psychological resilience. This pattern suggests that psychological assessment in dialysis settings should not be limited to symptom identification but should also consider adaptive capacity. Interventions aimed at strengthening psychological resilience, such as CBT, may help improve psychological adaptation and partially mitigate the adverse impact of anxiety, particularly in the mental health domains. Early screening and timely psychological intervention may therefore represent important components of comprehensive dialysis care. Second, insomnia requires targeted management strategies, including sleep hygiene education and possibly behavioral sleep therapy, as resilience enhancement alone may be insufficient. Third, the observed effect modification by smoking status and diabetes suggests that psychological distress may exert greater harm in patients with lifestyle-related and metabolic comorbidities. This highlights the need for stratified intervention strategies that integrate psychological care with metabolic and behavioral risk management in dialysis populations.

This study has several strengths. First, we simultaneously evaluated anxiety, insomnia, psychological resilience, and HRQoL within a unified mediation framework, allowing for quantification of indirect effects. Second, we employed the SF-36 to separately evaluate physical and mental health domains, enabling a clearer differentiation between psychological and somatic pathways. This dimensional structure was particularly suitable for mediation analysis examining resilience, as it allowed us to quantify distinct effects on PCS and MCS. Third, subgroup analyses enabled exploration of effect modification by key demographic and clinical variables, strengthening the robustness and clinical interpretability of the findings.

Nevertheless, several limitations should be noted. First, the cross-sectional design precludes causal inference; therefore, mediation findings should be interpreted cautiously as statistical rather than temporal mediation. The observed mediation relationships should be considered hypothesis-generating rather than causal. Longitudinal studies are warranted to confirm temporal ordering and directionality.

Second, this study was conducted at a single center with a relatively limited sample size, which may restrict the generalizability of the findings and reduce statistical power for detecting modest effects or interactions. In particular, patient characteristics, clinical practices, and psychosocial support systems may vary across institutions and regions, potentially limiting the external validity of the results. Multicenter studies with larger and more diverse populations are warranted to enhance generalizability. Third, patients with previously diagnosed psychiatric disorders were excluded to ensure participants were able to complete questionnaires reliably; however, this exclusion criterion may have introduced selection bias. Specifically, individuals with more severe psychological symptoms may have been underrepresented in the study population, which could lead to an underestimation of the strength of the associations between psychological factors and HRQoL. Therefore, the findings may be more generalizable to clinically stable dialysis patients rather than to those with significant psychiatric comorbidities. In addition, all psychological constructs were assessed using self-reported instruments, which may introduce reporting bias and shared method variance. Finally, although multiple demographic and clinical covariates were adjusted for, residual confounding from unmeasured or imperfectly measured factors, such as socioeconomic status, social support, medication adherence, or dialysis adequacy, cannot be entirely excluded.

## 5. Conclusions

In maintenance dialysis patients, anxiety and insomnia are independently associated with impaired health-related quality of life. Psychological resilience partially mediates the association between anxiety and HRQoL, but does not significantly mediate the relationship between insomnia and HRQoL. These findings highlight distinct psychological pathways and underscore the importance of tailored intervention strategies targeting both emotional distress and adaptive capacity in this vulnerable population.

## Data Availability

Data described in the manuscript, code book, and analytic code will be made available upon reasonable request.

## References

[1] GBD 2023 Chronic Kidney Disease Collaborators. Global, regional, and national burden of chronic kidney disease in adults, 1990-2023, and its attributable risk factors: a systematic analysis for the Global Burden of Disease Study 2023. Lancet. 2025;406(10518):2461–2482. doi: 10.1016/S0140-6736(25)01853-7. 41213283

[2] Herrington WG, Judge PK, Grams ME, et al. Chronic kidney disease. Lancet. 2026;407(10523):90–104. doi: 10.1016/S0140-6736(25)01942-7. 41314225

[3] Liyanage T, Ninomiya T, Jha V, et al. Worldwide access to treatment for end-stage kidney disease: a systematic review. Lancet. 2015;385(9981):1975–1982. doi: 10.1016/S0140-6736(14)61601-9. 25777665

[4] Brown EA, Zhao J, McCullough K, et al. Burden of kidney disease, health-related quality of life, and employment among patients receiving peritoneal dialysis and in-center hemodialysis: findings From the DOPPS Program. Am J Kidney Dis. 2021;78(4):489–500.e1. doi: 10.1053/j.ajkd.2021.02.327. 33872688

[5] Fletcher BR, Damery S, Aiyegbusi OL, et al. Symptom burden and health-related quality of life in chronic kidney disease: a global systematic review and meta-analysis. PLoS Med. 2022;19(4):e1003954. doi: 10.1371/journal.pmed.1003954. 35385471 PMC8985967

[6] Janssen IM, Gerhardus A, von Gersdorff GD, et al. Preferences of patients undergoing hemodialysis – results from a questionnaire-based study with 4,518 patients. Patient Prefer Adherence. 2015;9:847–855. doi: 10.2147/PPA.S79559. 26170634 PMC4492657

[7] Stenman U, Hakama M, Knekt P, et al. Measurement and modeling of health-related quality of life. Epidem Demog Public Health. 2010;195(1):130–135.

[8] Ware JE, Gandek B. Overview of the SF-36 Health Survey and the International Quality of Life Assessment (IQOLA) ProjectSF-36. J Clin Epidemiol. 1998;51(11):903–912. doi: 10.1016/S0895-4356(98)00081-X. 9817107

[9] Rincon Bello A, Ion Titapiccolo J, Berdud Godoy I, et al. Better health-related quality of life is associated with prolonged survival and reduced hospitalization risk among dialysis-dependent chronic kidney disease patients: a historical cohort study. BMC Nephrol. 2024;25(1):388. doi: 10.1186/s12882-024-03835-0. 39482605 PMC11526659

[10] Kraus MA, Fluck RJ, Weinhandl ED, et al. Intensive hemodialysis and health-related quality of life. Am J Kidney Dis. 2016;68(5S1):S33–S42. doi: 10.1053/j.ajkd.2016.05.023. 27772641

[11] Schouten RW, Haverkamp GL, Loosman WL, et al. Anxiety symptoms, mortality, and hospitalization in patients receiving maintenance dialysis: a cohort study. Am J Kidney Dis. 2019;74(2):158–166. doi: 10.1053/j.ajkd.2019.02.017. 31027882

[12] Lee MJ, Lee E, Park B, et al. Mental illness in patients with end-stage kidney disease in South Korea: a nationwide cohort study. Kidney Res Clin Pract. 2022;41(2):231–241. doi: 10.23876/j.krcp.21.047. 34974656 PMC8995483

[13] Preljevic VT, Østhus TBH, Os I, et al. Anxiety and depressive disorders in dialysis patients: association to health-related quality of life and mortality. Gen Hosp Psychiatry. 2013;35(6):619–624. doi: 10.1016/j.genhosppsych.2013.05.006. 23896282

[14] Chen S, Wang Y, Feng S, et al. Anxiety as a mediator between symptom distress and quality of life in peritoneal dialysis patients: insights from mediation analysis and nonlinear models. Ren Fail. 2025;47(1):2458763. doi: 10.1080/0886022X.2025.2458763. 39901459 PMC11795746

[15] Bujang MA, Musa R, Liu WJ, et al. Depression, anxiety and stress among patients with dialysis and the association with quality of life. Asian J Psychiatr. 2015;18:49–52. doi: 10.1016/j.ajp.2015.10.004. 26549864

[16] Lyons OD. Sleep disorders in chronic kidney disease. Nat Rev Nephrol. 2024;20(10):690–700. doi: 10.1038/s41581-024-00848-8. 38789686

[17] Iliescu EA, Coo H, McMurray MH, et al. Quality of sleep and health-related quality of life in haemodialysis patients. Nephrol Dial Transplant. 2003;18(1):126–132. doi: 10.1093/ndt/18.1.126. 12480970

[18] Sisto A, Vicinanza F, Campanozzi LL, et al. Towards a transversal definition of psychological resilience: a literature review. Medicina (Kaunas). 2019;55(11):745. doi: 10.3390/medicina55110745. 31744109 PMC6915594

[19] Xu Q, Qiu Y, Yi T, et al. Social support and family resilience among Chinese people receiving maintenance hemodialysis: a polynomial regression and response surface analysis explaining psychological resilience. Appl Psychol Health Well Being. 2024;16(4):1905–1920. doi: 10.1111/aphw.12569. 38924268

[20] Tsanasidis M, Kafkia T, Papoutsis D, et al. Resilience, pain self-efficacy and health-related quality of life in greek hemodialysis patients: a cross-sectional study. Int J Prev Med. 2025;16:9. doi: 10.4103/ijpvm.ijpvm_108_24. 40115137 PMC11925358

[21] González-Flores CJ, García-García G, Lerma A, et al. Resilience: a protective factor from depression and anxiety in mexican dialysis patients. Int J Environ Res Public Health. 2021;18(22):11957. doi: 10.3390/ijerph182211957. 34831713 PMC8620979

[22] Palagini L, Moretto U, Novi M, et al. Lack of resilience is related to stress-related sleep reactivity, hyperarousal, and emotion dysregulation in insomnia disorder. J Clin Sleep Med. 2018;14(5):759–766. doi: 10.5664/jcsm.7100. 29734989 PMC5940426

[23] Li L, Wang H, Shen Y. Development and psychometric tests of a Chinese version of the SF-36 Health Survey Scales. Zhonghua Yu Fang Yi Xue Za Zhi. 2002;36(2):109–113. 12410965

[24] Sung SA, Hyun YY, Lee KB, et al. Sleep duration and health-related quality of life in predialysis CKD. Clin J Am Soc Nephrol. 2018;13(6):858–865. doi: 10.2215/CJN.11351017. 29724791 PMC5989677

[25] Gong Y, Zhou H, Zhang Y, et al. Validation of the 7-item Generalized Anxiety Disorder scale (GAD-7) as a screening tool for anxiety among pregnant Chinese women. J Affect Disord. 2021;282:98–103. doi: 10.1016/j.jad.2020.12.129. 33401129

[26] Soldatos CR, Dikeos DG, Paparrigopoulos TJ. Athens Insomnia Scale: validation of an instrument based on ICD-10 criteria. J Psychosom Res. 2000;48(6):555–560. doi: 10.1016/s0022-3999(00)00095-7. 11033374

[27] Kawaratani H, Miyaaki H, Hiraoka A, et al. The usefulness of the athens insomnia scale for evaluating sleep disturbance in patients with chronic liver disease comparing with Pittsburgh Sleep Quality Index and Epworth Sleepiness Scale. Medicina (Kaunas). 2022;58(6):741. doi: 10.3390/medicina58060741. 35744004 PMC9229656

[28] Connor KM, Davidson JRT. Development of a new resilience scale: the Connor–Davidson Resilience Scale (CD-RISC). Depress Anxiety. 2003;18(2):76–82. doi: 10.1002/da.10113. 12964174

[29] Wu L, Tan Y, Liu Y. Factor structure and psychometric evaluation of the Connor–Davidson resilience scale in a new employee population of China. BMC Psychiatry. 2017;17(1):49. doi: 10.1186/s12888-017-1219-0. 28152997 PMC5290619

[30] Zhang Y, Zhang A, Xiang J, et al. Perceived stress and cardiovascular disease in a community-based population. Heart and Mind. 2022;6(4):262–266. doi: 10.4103/hm.hm_55_22.

[31] Cal SF, Sá LRd, Glustak ME, et al. Resilience in chronic diseases: a systematic review. Cogent Psychol. 2015;2(1):1024928. doi: 10.1080/23311908.2015.1024928.

[32] Sharma S, Shrivastava A, Singh A. Resilience in management of chronic diseases: a review of the strategies, approaches, and interventions. Discov Public Health. 2025;22(1):724. doi: 10.1186/s12982-025-01100-9.

[33] Li X, Gao W, Yu J, et al. Mental health and quality of life in chronic kidney disease patients with mild-to-moderate depression: a retrospective cohort study of mindfulness-based stress reduction therapy. Actas Esp Psiquiatr. 2024;52(2):138–148. doi: 10.62641/aep.v52i2.1600. 38622007 PMC11015819

[34] Edinger JD, Arnedt JT, Bertisch SM, et al. Behavioral and psychological treatments for chronic insomnia disorder in adults: an American academy of sleep medicine clinical practice guideline. J Clin Sleep Med. 2021;17(2):255–262. doi: 10.5664/jcsm.8986. 33164742 PMC7853203

[35] Jacobs GD, Pace-Schott EF, Stickgold R, et al. Cognitive behavior therapy and pharmacotherapy for insomnia: a randomized controlled trial and direct comparison. Arch Intern Med. 2004;164(17):1888–1896. doi: 10.1001/archinte.164.17.1888. 15451764

[36] Troy AS, Willroth EC, Shallcross AJ, et al. Psychological resilience: an affect-regulation framework. Annu Rev Psychol. 2023;74(1):547–576. doi: 10.1146/annurev-psych-020122-041854. 36103999 PMC12009612

[37] Cohen SD, Cukor D, Kimmel PL. Anxiety in patients treated with hemodialysis. Clin J Am Soc Nephrol. 2016;11(12):2250–2255. doi: 10.2215/CJN.02590316. 27660303 PMC5142059

[38] Broche-Pérez Y, Jiménez-Morales RM. Psychological resilience as a mediator between depression and health-related quality of life in relapsing-remitting multiple sclerosis patients. Mult Scler Relat Disord. 2026;106:106919. doi: 10.1016/j.msard.2025.106919. 41385982

[39] Hohls JK, König HH, Quirke E, et al. Anxiety, depression and quality of life –a systematic review of evidence from longitudinal observational studies. Int J Environ Res Public Health. 2021;18(22):12022. doi: 10.3390/ijerph182212022. 34831779 PMC8621394

[40] Riepenhausen A, Wackerhagen C, Reppmann ZC, et al. Positive cognitive reappraisal in stress resilience, mental health, and well-being: a comprehensive systematic review. Emotion Rev. 2022;14(4):310–331. doi: 10.1177/17540739221114642.

[41] Sousa H, Ribeiro O, Figueiredo D. Purpose in life among haemodialysis caregivers: links with adaptive coping, caregiver burden, and psychological distress. Stress Health. 2024;40(5):e3460. doi: 10.1002/smi.3460. 39134404

[42] Bulathwatta DT, Rudnik A, Borchet J, et al. Contrasting cultures, shared struggles: a qualitative analysis of the experiences of end-stage kidney disease patients and their caregivers in Poland and Sri Lanka. SAGE Open Nurs. 2025;11:23779608251360594. doi: 10.1177/23779608251360594. 40761615 PMC12319197

[43] Mm Z, Ar R, M R, et al. N M. The lived experience of resilience in chronic disease among adults in Asian countries: a scoping review of qualitative studies. BMC Psychol. 2024;12(1):773. doi: 10.1186/s40359-024-02296-2. PMC11663340 39710785

[44] Ali DA, Figley CR, Tedeschi RG, et al. Shared trauma, resilience, and growth: A roadmap toward transcultural conceptualization. Psychol Trauma. 2023;15(1):45–55. doi: 10.1037/tra0001044. 34138612

[45] Fernandez-Mendoza J, Vgontzas AN. Insomnia and its impact on physical and mental health. Curr Psychiatry Rep. 2013;15(12):418. doi: 10.1007/s11920-013-0418-8. 24189774 PMC3972485

